# SLCO3A1 expression is a major determinant of atazanavir PBMC penetration in HIV-infected patients

**DOI:** 10.1186/1758-2652-13-S4-P178

**Published:** 2010-11-08

**Authors:** M Siccardi, A D'Avolio, L Baietto, M Simiele, S Bonora, DJ Back, G Di Perri, A Owen

**Affiliations:** 1 University of Liverpool, Department of Pharmacology and Therapeutics, Liverpool, UK; 2 University of Turin, Turin, Italy; 3 University of Liverpool, Department of Molecular and Clinical Pharmacology, Liverpool, UK

## Background

Atazanavir (ATV) is administered at a dose of 300 mg boosted with 100 mg of ritonavir once daily (boosted). However, in the clinical setting use of unboosted ATV can be warranted. Although plasma concentrations are used clinically as a marker of drug exposure, ATV predominantly acts within HIV infected cells and therefore intracellular concentrations may better correlate with therapeutic efficacy. To date, the factors that define ATV flux into peripheral blood mononuclear cells (PBMC) are not fully characterized and several efflux and influx transporters may influence intracellular pharmacokinetics.

## Purpose of the study

To evaluate gene expression of efflux (ABCB1, ABCC1, ABCC2) and influx (SLCO3A1) transporters and relate to the cellular accumulation of unboosted ATV.

## Methods

Patients administered with unboosted ATV were recruited in Torino. Written informed consent was obtained. Main inclusion criteria were: no concomitant interacting drugs (except for TDF), no hepatic or renal impairment and self-reported adherence > 95%. Blood samples were collected 22-26 h after dosing and PBMC and plasma separated. Plasma samples were analysed by a validated HPLC-PDA method and PBMC extracts (intracellular) analysed using a validated LC-MS method. The cell count and mean cellular volume (MCV) was used for determining intracellular concentrations. Gene expression was evaluated by relative quantification using real time PCR.

## Results

13 Caucasian patients met the inclusion criteria and were included in the study. Median plasma ATV Ctrough was 134 ng/ml (IQR, 113-153), intracellular concentrations were 322 ng/ml (IQR, 210-448), and the median accumulation ratio (intracellular/plasma concentration) was 1.84 (IQR, 1.21–3.58). Intracellular concentrations were not correlated with plasma concentrations (rho= -0.22 p=0.41). SLCO3A1 expression was significantly correlated with cellular accumulation ratio. (rho=0.626, p=0.022). In multivariate linear regression analyses, SLCO3A1 expression was the only independent predictor of ATV cellular accumulation (â=0.726, p=0.007).

## Conclusions

Intracellular ATV concentrations were higher than plasma concentrations, indicating an accumulation of ATV in PBMC and potentially a role for influx transporters. The correlation between SLCO3A1 expression and ATV accumulation supports this hypothesis and suggests that the SLCO3A1 uptake transporter may be a determinant of intracellular ATV concentrations.

